# Adjunctive palivizumab with ribavirin for respiratory syncytial virus infection during the early peri-transplant period in adult allogenic hematopoietic stem cell transplant recipients: Case series

**DOI:** 10.1016/j.idcr.2026.e02721

**Published:** 2026-08-08

**Authors:** Latefah Aleshaiwi, Afrah Alotaibi, Mohsen Alzahrani, Mohammad Bosaeed, Hajar AlQahtani, Abdulellah Almohaya

**Affiliations:** a Infectious Diseases Division, Department of Medicine, King Abdulaziz Medical City, Riyadh, Saudi Arabia; b Department of Pharmaceutical Care, Ministry of National Guard Health Affairs, Riyadh, Saudi Arabia; c Department of Hematology/Oncology, King Abdulaziz Medical City, Ministry of National Guard Health Affairs, Saudi Arabia; d King Abdullah International Medical Research Center, Riyadh, Saudi Arabia; e King Saud Bin Abdul-Aziz University for Health Sciences, Riyadh, Saudi Arabia

**Keywords:** RSV, Respiratory infection, Stem Cell Transplant, Palivizumab, Monoclonal antibody

## Abstract

**Background:**

Respiratory syncytial virus (RSV) is associated with significant morbidity and mortality in hematopoietic stem cell transplant (HSCT) recipients, particularly during the peri-transplant period. Therapeutic options remain limited, and the role of adjunctive RSV-specific immunotherapy is unclear. We evaluated the outcomes of palivizumab combined with ribavirin in high-risk adult HSCT recipients with RSV infection.

**Methods:**

We retrospectively reviewed adult HSCT recipients with RT-PCR-confirmed RSV infection who received palivizumab and ribavirin. Clinical characteristics, treatment timing, outcomes, adverse events, and mortality were assessed.

**Results:**

Three adult allogeneic HSCT recipients who developed RSV infection between (day – 4 to day +2) were identified. Two patients survived, who had moderate- to high-risk disease according to the Immunodeficiency Scoring Index (ISI-RSV), while one patient with high-risk disease developed progressive respiratory failure and died despite intensive therapy, likely due to concomitant mucormycosis. No treatment-related adverse events were observed. Earlier initiation of ribavirin and palivuzumab, and the intravenous route of palivuzumab, were observed in the two cases who achieved complete recovery.

**Conclusions:**

Adjunctive palivizumab appears to be safe and may be a useful therapeutic option for HSCT recipients with RSV infection during the peri-transplant period. Larger prospective studies are needed to define the optimal role, timing, and route of palivizumab administration in this high-risk population.

## Introduction

Respiratory syncytial virus (RSV) infection represents a significant cause of morbidity and mortality in immunocompromised patients, particularly among hematopoietic stem cell transplant (HSCT) recipients during the early peri-transplantation period. The cumulative incidence of RSV infection in allogeneic HSCT recipients approaches 62% within the first year post-transplant, with progression to lower respiratory tract disease (LRTD) occurring in 7.6–39% of cases [1], [2]. RSV-associated mortality at 90–100 days post-detection ranges from 5.4% to 8.7% and increasing to 18% among patients who develop LRTD [1], [3]. Beyond acute mortality, RSV infection is associated with long-term pulmonary sequelae, including bronchiolitis obliterans syndrome and irreversible airflow obstruction, which contribute to substantial late mortality [2], [4].

The early peri-transplantation period represents a window of heightened vulnerability due to profound immunosuppression, lymphopenia, neutropenia, and the absence of protective adaptive immunity. Risk stratification tools such as the Immunodeficiency Scoring Index (ISI) and the Basel Severe Immunodeficiency grading have been developed to identify patients at the highest risk for progression to LRTD and death [1], [5]. High-risk ISI scores (7−12) are associated with four-fold increased risk of LRTD progression and significantly elevated mortality [1], [4].

Current therapeutic options for RSV in immunocompromised adults remain limited. Ribavirin, a nucleoside analog with broad antiviral activity, has been incorporated into selective management recommendations for adult allogeneic HSCT recipients, based on uncontrolled observational data [5]. Recent meta-analyses suggest that ribavirin may reduce all-cause mortality in patients with LRTD (pooled OR 0.19, 95% CI 0.07–0.51) and decrease progression to LRTD when administered as aerosolized therapy (pooled OR 0.27, 95% CI 0.09–0.80), though the certainty of evidence remains low to moderate [6]. Current guidelines recommend oral or intravenous ribavirin at 10–30 mg/kg/day (maximum 1800 mg/day) for allogeneic HSCT recipients with LRTD or those at high risk for progression, with prompt initiation at the early stage of upper respiratory tract infection viewed as potentially key to efficacy [5].

Palivizumab, a humanized monoclonal antibody targeting the RSV fusion protein, has been approved since 1998 for prophylaxis in high-risk pediatric populations but has limited data supporting its use in adults [7]. Its use in RSV prophylaxis has dramatically decreased after the approval of Nirsevimab, which provides expansion of use, longstanding protection, and lower cost [8], [9]. Current expert consensus from the 10th European Conference on Infections in Leukaemia discourages the use of palivizumab as primary, pre-exposure, or post-exposure prophylaxis in adults with hematological malignancies or undergoing HCT [5]. However, some experts consider palivizumab for severely immunosuppressed, hospitalized patients during nosocomial outbreaks, and its potential role as adjunctive therapy in combination with ribavirin for established RSV infection in high-risk adult patients remains incompletely defined [5].

Due to the critical outcome of the peri-transplant period, lack of established treatment protocols, the limited RSV specific antibody within commercially available IVIG, the addition of palivizumab was considreed and warranted investigations. The rationale for combination therapy with palivizumab and ribavirin is based on complementary mechanisms of action: ribavirin inhibits viral RNA synthesis, while palivizumab provides passive immunotherapy by neutralizing extracellular virus and preventing cell-to-cell spread.

This case series describes the clinical charactastistics of adult HSCT recipients who received adjunctive palivizumab with ribavirin and IVIG for RSV infection occurring during the early peri-transplantation period, a population at particularly high risk for severe disease and death.

## Methods

### Study design and setting

This retrospective case series evaluated adult stem cell transplant recipients who received combination therapy with palivizumab and ribavirin for respiratory syncytial virus (RSV) infection during the early peri-transplantation period. Cases were identified from the tertiary transplant center, King Abdulaziz Medical City, Riyadh, Saudi Arabia, between January 2023 to June 2026.

### Patient selection and data collection

Eligible patients included adults (≥18 years) who underwent allogeneic or autologous hematopoietic stem cell transplantation and developed laboratory-confirmed RSV infection between 1 week prior to cell infusion and engraftment. Patients who received combination palivizumab and ribavirin therapy were included. Patients were excluded if they had incomplete medical records or if RSV infection occurred outside the defined peri-transplantation period. Extracted data included patient and transplant characteristics, immunosuppression, laboratory and RSV infection parameters, treatments, adverse events, clinical outcomes, and 30- and 90-day mortality.

### RSV diagnosis and classification

RSV infection was confirmed by reverse transcription polymerase chain reaction (RT-PCR) from nasopharyngeal swab or bronchoalveolar lavage. Infections were classified as upper respiratory tract infection (URTI) or lower respiratory tract infection (LRTI) based on clinical and radiographic findings. LRTI was defined as the presence of new pulmonary infiltrates on chest imaging with respiratory symptoms and/or hypoxemia.

### Risk stratification

Patients were stratified by the immunodeficiency severity index that was validated to predict poor outcomes in hemoatopoietic cell transplant [10]. It consists of 7 elements with each with assigned weights between 1 and 3 points. High-risk features included absolute neutrophil counts < 500 cells/μL (3 points), lymphocyte count < 200 cells/μL (3 points), age > = 40 years (2 points), myeloablative conditioning regimen (1 point), graft versus host diseases (GVHD) (1 point), corticosteroid (1 point), and recent or pre-engraftment allo-SCT (1 point).

### Treatment protocol

Ribavirin Administration: Ribavirin was administered orally at a dose of 10–30 mg/kg body weight in three divided doses, maximum 600 mg every 8 h, total daily dose up to 1800 mg. Treatment duration was determined by clinical response and ranged from 7 to 21 days.

Palivizumab Administration: Palivizumab was administered at a dose of 15 mg/kg via intramuscular injection or intravenous route, at single or repeated doses. The actual received dose is written in the case section.

Adjunctive Therapy: Intravenous immunoglobulin (IVIG) was administered at 0.5 g/kg bodyweight for patients with hypogammaglobulinemia or at high risk for progression to LRTI. Up to five doses were prescribed according to the discretion of the treating physician. Supportive care included bronchodilators, supplemental oxygen, intravenous fluids, and antipyretics as clinically indicated.

### Outcome measures and safety monitoring

Primary outcomes included clinical response (resolution of respiratory symptoms and radiographic findings) and all-cause mortality at 30 and 90 days. Secondary outcomes included viral clearance and adverse events related to therapy. Ribavirin-related toxicities and drug interactions were monitored daily during hospitalization and at outpatient follow-up, with dose adjustment or discontinuation based on toxicity severity. Follow up for survived patients was extended until the last day of the study.

### Cases (Summarized in Table 1)

#### Case−1

A 30-year-old man with sickle cell disease underwent a matched related donor (MRD) allogeneic stem cell transplantation (allo-SCT) for recurrent vaso-occlusive crises (VOC), sickle cell hepatopathy, and a prior history of stroke. He was admitted during the month of January for planned conditioning. At admission, respiratory viral screening by quadriplex nasopharyngeal aspirate (NPA) PCR was negative for respiratory syncytial virus (RSV).

**Table 1:** Summary of reported case of Pavilizumab adjunct use with Ribavirin among Stem Cell Transplant Patients (n = 3).

	Case−1	Case−2	Case−3
Host	30 year old male with MRD-Allo-SCT, for SCD	19 year old male with Haplo-SCT, for SCD	19 year old female with Haplo-SCT, for SCD
Height/Weight	183 cm, 81 kg	166 cm, 80 kg	152 cm, 38 kg
Conditioning Regimen	Alemtuzumab, TBI	ATG, Thiotepa, Cyclocphosphamide	ATG, Thiopeta, Fludaribine, Cyclophosphamide, TBI
GVHD Prophylaxis	Sirolimus	Sirolimus	MMF, Sirolimus
Engrafement Day	D + 17	D + 25	Not engrafted
Timing of infection (RSV PCR Positive)	Day – 3	Day + 2	Day – 4
Ribavirin Dose	400 mg BID (10 mg/kg/day)	600 mg PO TID (maximum dose)	200 mg PO TID (15 mg/kg/day)
Ribavirin Duration (days)	7 days	10 days	21 days
Palivuzumab dose	1000 mg IV (12 mg/kg)	1200 mg IV (15 mg/kg)	600 mg IM (15 mg/kg)
Disease stage at Palivizumab administration	URTI	URTI	LRTI
Safety (any reported allergy or side effects)	None	None	None
ISI-RSV Score:	4/12 (Moderate)	8/12 High	8/12 (High)
− ANC < 0.500	16.7 (0/3)	0.0 (3/3)	0.0 (3/3)
− ALC < 0.200	0.0 (3/3)	0.0 (3/3)	0.0 (3/3)
− Age > 40 years	No (0/2)	No (0/2)	No (0/2)
− MA regimen	No (0/1)	Yes (1/1)	Yes (1/1)
− GVHD	No (0/1)	No (0/1)	No (0/1)
− Steroid within 30 days	No (0/1)	No (0/1)	No (0/1)
− Pre-engraftment or recent engraftment within 30 days	Yes (1/1)	Yes (1/1)	Yes (1/1)
Symptom’s onset or PCR positive to treatment:			
− To RBV	2 days	2 days	3 days
− To IVIG	2 days	4 days	8 days
− To Palivizumab	6 days	4 days	8 days
Hospital Length of Stay	14 days	92 days	38 days
Outcome RSV PCR	No repeat	Persistent shedding	Persistent shedding
Outcome ICU admission	No	No	Yes
Outcome Survival at 30 days	Survived	Survived	Died D + 40 due to pulmonary mucormycosis

* MRD: matched-related donor. SCT: Stem cell transplant. SCD: Sickle cell disease. TBI: total body irradiation. ATG: Anti-thymocyte Globulin. GVHD (Graft-versus-Host Disease), MMF (Mycophenolate Mofetil), RSV (Respiratory Syncytial Virus), PCR (Polymerase Chain Reaction), BID (twice daily), PO (oral administration), TID (three times daily), IV (intravenous), IM (intramuscular), URTI (Upper Respiratory Tract Infection), LRTI (Lower Respiratory Tract Infection), ISI-RSV (Immunodeficiency Scoring Index for RSV), ANC (Absolute Neutrophil Count), ALC (Absolute Lymphocyte Count), MA (Myeloablative), RBV (Ribavirin), IVIG (Intravenous Immunoglobulin), ICU (Intensive Care Unit), D + (days after transplantation).

On day 2 of admission, conditioning was initiated with alemtuzumab for 5 days, in addition to total body irradiation (TBI) and sirolimus. On the final day of alemtuzumab administration, he developed a dry cough, sore throat, fever, and shortness of breath. Despite these symptoms, he remained clinically stable on room air without respiratory distress. Respiratory viral panel PCR performed on the same day was positive for RSV (transplant day – 3). Laboratory evaluation showed a white blood cell count of 17.3 × 10⁹/L, and chest radiography was unremarkable. His ISI-RSV score was 4 out of 12 (moderate risk of progression).

Treatment with oral ribavirin 600 mg thrice a day was initiated on transplant day −1 (admission day 9) and continued for 7 days, together with intravenous immunoglobulin (IVIG) 10% at a dose of 30 g daily for 4 days. Allogeneic stem cell infusion was performed on transplant day 0 (admission day 10). A single dose of palivizumab 1000 mg (intravenous) was subsequently administered on transplant day + 2 (admission day 12; infection day 6).

During follow-up, the patient's respiratory symptoms gradually improved. He remained afebrile, maintained adequate oxygenation on room air, and demonstrated stable respiratory and hemodynamic status throughout hospitalization. He was discharged two weeks later in good clinical condition and continues to be followed regularly without significant complications to date.

#### Case−2

A 19-year-old man with sickle cell disease underwent haploidentical allogeneic stem cell transplantation (haplo-allo-SCT) for recurrent episodes of acute chest syndrome. He was electively admitted in the month of December for transplantation and began conditioning on admission day 2 with antithymocyte globulin (ATG), thiotepa, and cyclophosphamide. Stem cell infusion was performed on admission day 9 (transplant day 0), after which sirolimus was initiated for graft-versus-host disease prophylaxis.

On transplant day + 2, he developed febrile neutropenia accompanied by productive cough, abdominal pain, and diarrhea. He remained hemodynamically stable and maintained adequate oxygenation on room air throughout this period. Diagnostic workup revealed a positive respiratory viral panel for respiratory syncytial virus (RSV), while gastrointestinal multiplex PCR was positive for Salmonella species. Blood cultures remained negative, and chest radiography showed no acute abnormalities. Empiric antimicrobial therapy with meropenem and vancomycin was initiated. The ISI-RSV score was 8 out of 12 (high risk of progression).

On transplant day + 4, oral ribavirin 600 mg twice daily was added and continued for 10 days. Adjunctive therapy included a single dose of palivizumab administered on transplant day + 6, along with daily intravenous immunoglobulin (IVIG) for four consecutive doses.

Over the subsequent days, both respiratory and gastrointestinal symptoms gradually improved, and the fever resolved by transplant day + 8 despite ongoing neutropenia. Repeat quadriplex PCR on transplant day + 9 remained positive for RSV, with a cycle threshold (Ct) value of 24.7. Neutrophil engraftment occurred around transplant day + 24; however, persistent RSV shedding was documented until transplant day + 43, at which time the Ct value had increased to 30.

The remainder of his hospitalization was complicated by hospital-acquired pneumonia, possible invasive fungal pulmonary infection, and hemorrhagic cystitis requiring treatment with cidofovir. He was ultimately discharged in good clinical condition after a 92-day hospital stay and continues to be followed regularly in the outpatient clinic with a favorable clinical outcome to date.

#### Case−3

A 19-year-old woman with sickle cell disease was admitted in the month of September for haploidentical allogeneic stem cell transplantation (haplo-allo-SCT) due to recurrent acute chest syndrome. Admission RSV screening PCR was negative (transplant day −9), and conditioning was initiated with ATG, thiotepa, fludarabine, and cyclophosphamide.

On transplant day −4, she developed cough and shortness of breath and was diagnosed with RSV infection based on positive PCR from nasopharyngeal swab, in addition to concurrent Clostridioides difficile and enterotoxigenic Escherichia coli (ETEC) gastrointestinal infections. The ISI-RSV score was 8 (high risk of progression), and hence oral ribavirin was initiated on transplant day −1; the dose was ribavirin 200 mg every 8 h for 7 days.

**Fig. 1:**
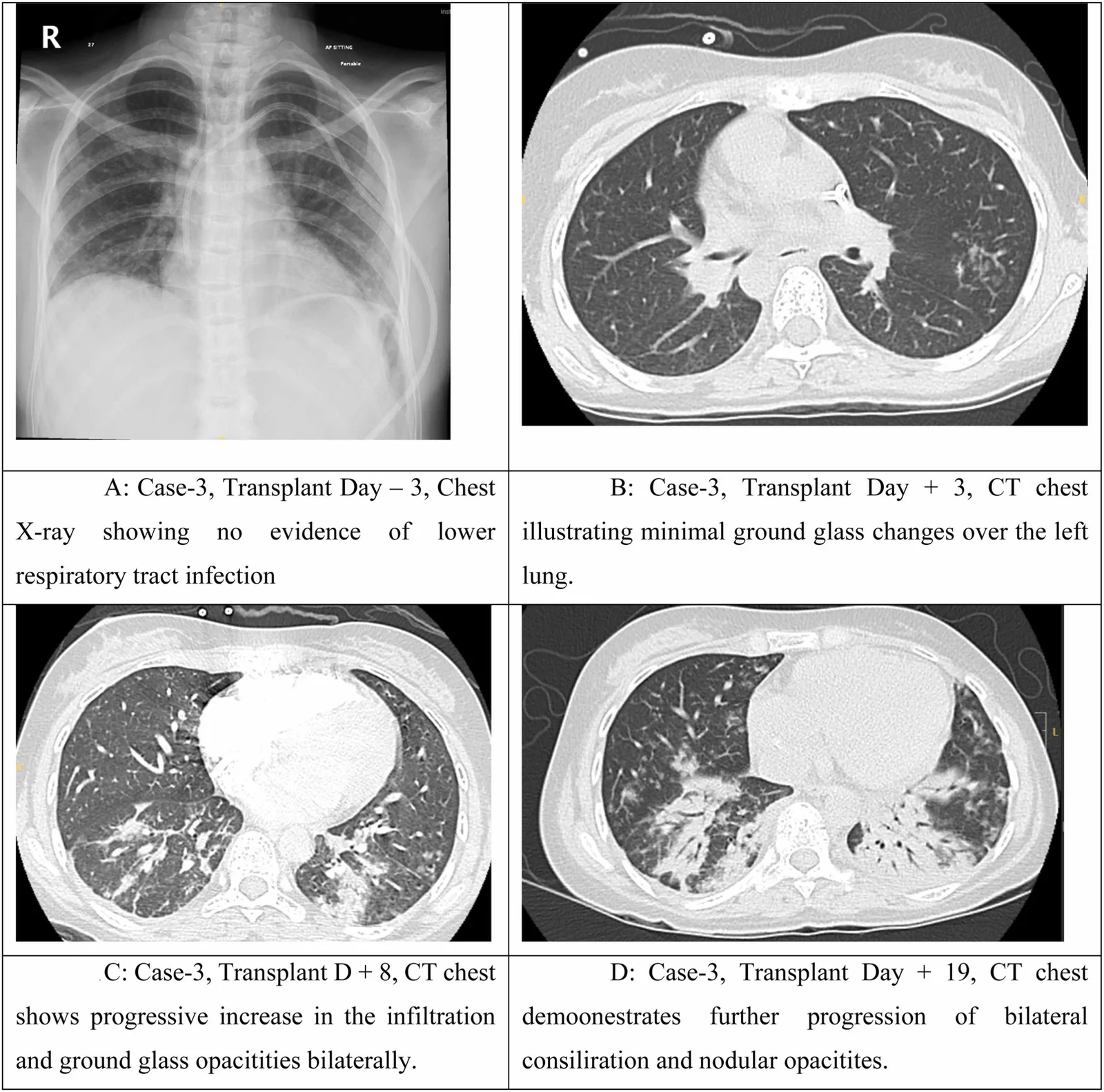
A: Case−3, Transplant Day – 3, Chest X-ray showing no evidence of lower respiratory tract infection, B: Case−3, Transplant Day + 3, CT chest illustrating minimal ground glass changes over the left lung. C: Case−3, Transplant D + 8, CT chest shows progressive increase in the infiltration and ground glass opacitities bilaterally. D: Case−3, Transplant Day + 19, CT chest demoonestrates further progression of bilateral consiliration and nodular opacitites.

Following stem cell infusion, her post-transplant course was complicated on transplant day + 1 by febrile neutropenia, persistent diarrhea, and progressive respiratory symptoms. Initial imaging demonstrated mild bilateral pulmonary infiltrates and pansinusitis without evidence of invasive fungal disease (Fig. 1A & 1B). Despite broad-spectrum antimicrobial therapy, fever and cough worsened, prompting treatment with IVIG and intramuscular palivizumab on transplant day + 4. Fever resolved by transplant day + 5, and her symptoms initially improved.

However, by transplant day + 8, she developed worsening cough and new oxygen requirements, accompanied by radiologic progression of bilateral pulmonary infiltrates and worsening pansinusitis (Fig. 1C & D). Antimicrobial and antifungal therapy were escalated, including liposomal amphotericin B.

On transplant day + 12, she developed foul-smelling nasal discharge, and MRI findings raised concern for invasive fungal sinusitis. Prompt endoscopic examination for the sinuses by ENT showed no compelling evidence of invasive fungal infection, but biopsies for culture were obtained. Due to that, further empiric escalation of anibacterial coverage (ceftazidime-Avibactam + Amikacin).

Her condition further deteriorated by transplant day + 19, requiring intensive care admission and high-flow nasal oxygen support. Bronchoscopy was declined by the patient, and evaluation suggested a hyperinflammatory syndrome with markedly elevated ferritin and interleukin−6 levels, for which she received methylprednisolone and tocilizumab, resulting in transient clinical improvement and was sent back to the floor. Ribavirin was subsequently extended three weeks duration.

**Fig. 2:**
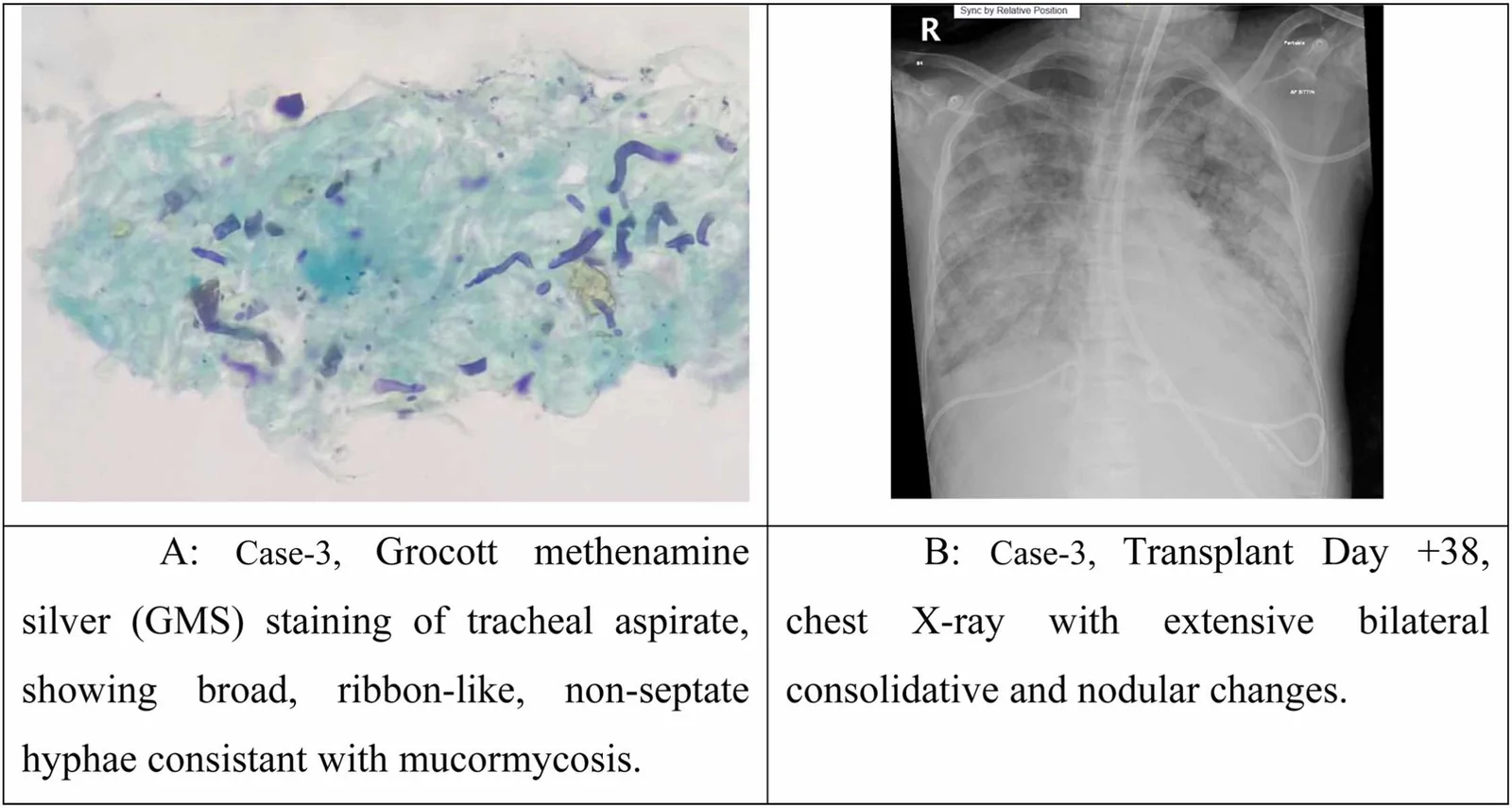
A: Case−3, Grocott methenamine silver (GMS) staining of tracheal aspirate, showing broad, ribbon-like, non-septate hyphae consistant with mucormycosis. B: Case−3, Transplant Day + 38, chest X-ray with extensive bilateral consolidative and nodular changes.

On transplant day + 26, she experienced recurrent rapid respiratory failure requiring re-admission to the intensive care unit, high-flow oxygen therapy, and eventual mechanical ventilation. Subsequent tracheal aspirate fluid revealed fungal elements on respiratory cytology consistent with mucormycosis (Fig. 2A & B), despite ongoing liposomal amphotericin B therapy, leading to the addition of isavuconazole. Fungal culture subsequently came back negative. Cardiac evaluation demonstrated reduced left ventricular systolic function, raising concern for myocarditis. Persistent RSV positivity in the setting of delayed engraftment prompted additional treatment with palivizumab and IVIG (second course).

Despite maximal supportive care and broad antimicrobial treatment, her respiratory failure progressed, and she died on transplant day + 38, approximately six weeks after RSV diagnosis.

## Discussion

This case series describes three allogeneic HSCT recipients who developed RSV infection during the peri-transplant period and received combination therapy with oral ribavirin, IVIG, and palivizumab. No safety concerns were identified. Two patients with moderate- to high-risk disease according to the Immunodeficiency Scoring Index (ISI-RSV) achieved complete recovery, while one patient developed progressive respiratory failure and died despite aggressive therapy, likely due to mucormycosis. The heterogeneous clinical outcomes observed in these three cases—ranging from uncomplicated recovery to prolonged viral shedding and fatal respiratory failure—suggest that prognosis is determined primarily by host factors rather than RSV infection alone, despite all patients receiving identical combination therapy with oral ribavirin, IVIG, and palivizumab.

The adjunctive use of IVIG in respiratory viral infections remains controversial. Although IVIG and palivizumab have been employed in high-risk transplant recipients based on theoretical immunologic benefits, clinical evidence supporting their routine use is limited. All three patients in this series received IVIG and palivizumab in addition to ribavirin, hence the additional benefit of palivizumab could not be assessed.

The evidence regarding palivizumab as adjunctive treatment for RSV infection in immunocompromised patients remains limited and conflicting. In pediatric populations, clinical trials in heterogeneous groups showed no additional benefit, though studies in high-risk populations, including children with leukemia, demonstrated survival benefits [11], [12]. Among adults, palivizumab has been used successfully for RSV prevention during institutional outbreaks in HSCT recipients without safety concerns [13]. Additionally, palivizumab was initially invetigated as a therapeutic option in a phase−1 clinical trial in 2001 among pediatric and adult stem cell transplant recipients with no safety signal [14].

Several factors appeared to distinguish the two patients who recovered from the patient who ultimately died, although RSV infection occurred during the pre-engraftment period in all three cases. First factor, RSV-directed therapy was initiated earlier in the survivors. The interval from symptom onset or diagnosis to treatment was shorter for ribavirin (2 days vs. 3 days), IVIG (2–4 days vs. 8 days), and palivizumab (4 days vs. 8 days) in the surviving patients compared with the fatal case. While definitive conclusions cannot be drawn from such a small series, these observations raise the possibility that earlier initiation of RSV-specific therapy may contribute to improved outcomes, particularly in highly immunocompromised patients undergoing allogeneic HSCT. [5]

The second factor, the stage of RSV disease at the time of adjunctive therapies. The fatal case received palivizumab after already developing lower respiratory tract infection signs on chest imaging, while the two surviving cases still had upper respiratory tract infection. This would be consistent with the general principle that earlier intervention is more effective in viral respiratory infections, although it cannot be distinguished from differences in baseline disease severity in this series.

Third Factor, the route of palivizumab. While the FDA labeling among the pediatric age group remain as intramuscular [15], several case reports have successfully used the intervenous route among RSV infection [12], [13]. Interstingly, the two surviving cases have received the intravenous route, while the fatal case received the intramuscular route. This theoretically could be explained by the rapid nature of IV infusion and hence rapid effect, but it requires further study [16]. However, this factor remains speculative given the small number of cases. Fourth factor, the development of mucomycosis in the fatal case could explain the unfortunate outcome.

It is worth mentioning that the current descriptive nature of these cases these findings are exploratory and hypothesis-generating rather than evidence supporting earlier therapy or one administration route over another.

Moreover, these cases also highlight the challenge of interpreting RSV-directed therapies in HSCT recipients. Although ribavirin remains the most commonly utilized antiviral agent for RSV in transplant populations, evidence supporting its efficacy is derived largely from retrospective studies and observational cohorts [5]. Several studies suggest that early ribavirin therapy may reduce progression from URTI to LRTI and improve survival in high-risk patients, particularly when administered before respiratory failure develops [9]. However, optimal dosing, route of administration, and treatment duration remain uncertain. In our series, all patients received oral ribavirin, reflecting its practicality and increasing adoption as an alternative to aerosolized formulations.

Importantly, palivizumab was well tolerated in all three patients (two were intravanous route), with no infusion-related reactions, hypersensitivity events, or other treatment-attributable adverse effects observed following administration. These observations suggest that adjunctive palivizumab can be administered safely in highly immunocompromised HSCT recipients, although larger studies are needed to better characterize its safety and efficacy in this setting. To our knowledge, reports describing adjunctive palivizumab administered during the immediate peri-transplant period (day −7 to day +7) in adult allogeneic HSCT recipients are extremely limited.

This case series has several limitations. The small number of patients limits generalizability, the lack of comparative control, and the observational nature of the report precludes assessment of treatment efficacy. Moreover, the lack of viral load monitoring and objective immunophenotyping response impairs direct observation of the impact of monoclonal antibody. In addition, multiple concurrent infections and transplant-related complications, particularly in the third patient, make it difficult to determine the independent contribution of RSV to outcomes. Nevertheless, these cases provide real-world insight into the spectrum of RSV infection among patients undergoing allogeneic HSCT and highlight challenges encountered in contemporary transplant practice.

## Conclusion

In conclusion, the use of palivizumab could serve as an adjunct therapy in heavily immunosuppressed patients, particularly in the early post-stem cell transplantation. We did not note a significant signal of safety. Further prospective studies are needed to better define optimal management strategies, explore the role of intravenous rather than intramuscular palivizumab, and the role of adjunctive therapies in HSCT recipients with RSV infection.

## CRediT authorship contribution statement

**Abdulellah Almohaya:** Writing – review & editing, Writing – original draft, Supervision, Methodology, Data curation, Conceptualization. **Hajar AlQahtani:** Writing – review & editing, Writing – original draft, Supervision, Methodology, Data curation, Conceptualization. **Mohammad Bosaeed:** Writing – review & editing, Writing – original draft, Supervision, Methodology, Data curation, Conceptualization. **Mohsen Alzahrani:** Writing – review & editing, Writing – original draft, Supervision, Methodology, Data curation, Conceptualization. **Afrah Alotaibi:** Writing – review & editing, Writing – original draft, Data curation, Conceptualization. **Latefah Aleshaiwi:** Writing – review & editing, Writing – original draft, Methodology, Data curation, Conceptualization.

## Ethics declaration

The authors have NOT obtained informed consent from participants or their legal representatives. Because no identifiable information was used. As per the IRB approval, Explicit authorization/consent from the patient must be sought for the use of identifiable information.

This study was performed in compliance with relevant laws, regulatory frameworks and guidelines where the research took place. This study was approved by the the King Abdullah International Medical Research Center (Approval No. 342026/ NRR26/036/5).

This research follows the CARE guidelines and the CARE checklist.

## Declaration of Competing Interest

The authors declare that they have no known competing financial interests or personal relationships that could have appeared to influence the work reported in this paper.
